# Bedside prediction of intradialytic hemodynamic instability in critically ill patients: the SOCRATE study

**DOI:** 10.1186/s13613-020-00663-x

**Published:** 2020-04-22

**Authors:** Naïke Bigé, Jean-Rémi Lavillegrand, Julien Dang, Philippe Attias, Stéphanie Deryckere, Jérémie Joffre, Vincent Dubée, Gabriel Preda, Guillaume Dumas, Geoffroy Hariri, Claire Pichereau, Jean-Luc Baudel, Bertrand Guidet, Eric Maury, Pierre-Yves Boelle, Hafid Ait-Oufella

**Affiliations:** 1 Service de Médecine Intensive Réanimation, AP-HP, Hôpital Saint-Antoine, Assistance Publique-Hôpitaux de Paris, 184 rue du Faubourg Saint-Antoine, Paris, 75571 Cedex 12 France; 2 Sorbonne Universités, Université Pierre et Marie Curie, Paris, 75006 France; 3 Inserm U1136, Paris, France; 4 Inserm U970, Paris Research Cardiovascular Center, Paris, France

**Keywords:** Hemodialysis, Acute kidney injury, Hemodynamic instability, Tissue perfusion, Lactate, Capillary refill time, SOFA score

## Abstract

**Background:**

Despite improvements in intermittent hemodialysis management, intradialytic hemodynamic instability (IHI) remains a common issue that could account for increased mortality and delayed renal recovery. However, predictive factors of IHI remain poorly explored. The objective of this study was to evaluate the relationship between baseline macrohemodynamic, tissue hypoperfusion parameters and IHI occurrence.

**Methods:**

Prospective observational study conducted in a 18-bed medical ICU of a tertiary teaching hospital. Cardiovascular SOFA score, index capillary refill time (CRT) and lactate level were measured just before (T0) consecutive intermittent hemodialysis sessions performed for AKI. The occurrence of IHI requiring a therapeutic intervention was recorded.

**Results:**

Two hundred eleven sessions, corresponding to 72 (34%) first sessions and 139 (66%) later sessions, were included. As IHI mostly occurred during first sessions (43% vs 12%, *P* < 0.0001), following analyses were performed on the 72 first sessions. At T0, cardiovascular SOFA score ≥1 (87% vs 51%, *P* = 0.0021) was more frequent before IHI sessions, as well as index CRT ≥ 3 s (55% vs 15%, *P* = 0.0004), and hyperlactatemia > 2 mmol/L (68% vs 29%, *P* = 0.0018). Moreover, the occurrence of IHI increased with the number of macrohemodynamic and tissue perfusion impaired parameters, named SOCRATE score (cardiovascular SOFA, index CRT and lactATE): 10% (95% CI [3%, 30%]), 33% (95% CI [15%, 58%]), 55% (95% CI [35%, 73%]) and 80% (95% CI [55%, 93%]) for 0, 1, 2 and 3 parameters, respectively (AUC = 0.79 [0.69–0.89], *P* < 0.0001). These results were confirmed by analyzing the 139 later sessions included in the study.

**Conclusions:**

The SOCRATE score based on 3 easy-to-use bedside parameters correlates with the risk of IHI. By improving risk stratification of IHI, this score could help clinicians to manage intermittent hemodialysis initiation in critically ill AKI patients.

## Background

According to international consensus definitions, acute kidney injury (AKI) concerns 30 to 40% of patients admitted to intensive care unit (ICU) [1–3]. Renal replacement therapy (RRT) is required in 5 to 20% of patients [1, 2] and is associated with high mortality [2, 4]. Despite the improvement of intermittent hemodialysis management [5, 6], intradialytic hemodynamic instability (IHI) remains a common issue [5, 7–12] that could account for increased mortality and delayed renal recovery. Optimal timing to initiate RRT in critically ill patients is still uncertain [4, 13, 14]. Apart from life-threatening complications, an international survey reported that indications for initiating RRT varied widely among intensivists [15]. Recent interventional trials demonstrated that delaying RRT in the absence of life-threatening complications of AKI does not impair survival and allows some patients to avoid RRT [4, 13]. Determination of risk factors for IHI could help clinicians to identify patients in whom initiation of RRT should be reconsidered in the absence of emergent criteria. However, factors associated with IHI remain poorly explored [8–12]. The pathophysiology of IHI is complex with a reduction of venous return and also an alteration of endothelial function that limits vascular tone adaptation to intermittent hemodialysis-induced hypovolemia [12, 16–18]. Pre-existing endothelial dysfunction, commonly observed in ICU patients [19–23], might worsen the negative hemodynamic impact of intermittent hemodialysis.

The evaluation of endothelial dysfunction at the bedside is very challenging, but its consequences on tissue hypoperfusion could be assessed more easily [24–26]. The prognosis value of lactate level and lactate clearance has been largely demonstrated [27, 28] and a therapeutic strategy targeting lactate normalization is recommended during sepsis and septic shock [29]. Capillary refill time (CRT) is an easy-to-use bedside [30] marker of peripheral perfusion. Its relevance has been demonstrated in triage of patients in pre-hospital setting [31] and in the emergency department [32, 33]. CRT correlates with severity of organ failure and is strongly associated with prognosis during septic shock [34]. Moreover, a recent multicenter study underlined the interest of a resuscitation strategy based on CRT monitoring in septic shock patients [35].

We hypothesized that critically ill patients with altered hemodynamics and/or impaired tissue perfusion might be more prone to develop IHI. In this prospective study, we tested whether cardiovascular SOFA score, index CRT and lactate level could be predictive of IHI requiring therapeutic intervention. The analyses led to the construction of a bedside score predictive of IHI.

## Methods

### Study population

We conducted a prospective observational study in the 18-bed medical ICU of a tertiary teaching hospital. During a 2-year period, all consecutive sessions of intermittent hemodialysis for AKI were recorded. Exclusion criteria were the following: dark skin because assessment of CRT was difficult, chronic intermittent hemodialysis and life-threatening complications such as hyperkalemia, and pulmonary edema in anuric patients indicating extreme emergency RRT.

### Intermittent hemodialysis management

In the absence of strong evidence supporting the use of one modality of renal replacement therapy over the other, and in accordance with European [36] and French guidelines [5, 6], intermittent hemodialysis is the only technique used in our ICU. Intermittent hemodialysis was prescribed according to national [5, 6] and international guidelines [36] in order to optimize hemodynamic tolerance. Both lines of the circuit filled with 0.9% saline were connected simultaneously to the catheter. Bicarbonate buffered dialysis solution with a calcium concentration of 1.75 mmol/L was used. Dialysate flow rate was set at 500 mL/min. Blood flow rate was progressively increased from 100 mL/min to 200–250 mL/min. For patients with pre-existing hemodynamic instability, dialysate temperature was set 2 degrees under patient’s body temperature and dialysate sodium concentration was set at ≥ 145 mmol/L. Fluid removal, if required, was started 60 min after starting intermittent hemodialysis. Intermittent hemodialysis was performed on the GAMBRO^®^ AK 200™ ULTRA S machine using NIPRO^®^ ELISIO™-13M dialyzer.

### Hemodynamic and tissue perfusion parameters collection

For each intermittent hemodialysis session, cardiovascular SOFA score, lactate level and index CRT were recorded just before starting the session (T0). Cut-off values defining tissue hypoperfusion were based on previously published studies: index CRT ≥ 3 s [26, 34, 35] and lactate level > 2 mmol/L [26, 29, 35]. Index CRT was measured in a standardized fashion as described before [34].

### Intradialytic hemodynamic instability

As no consensual definition of IHI exists in the literature [11], we chose to use a pragmatic definition, as previous authors [9]. IHI was defined as a blood pressure drop requiring therapeutic intervention, i.e., fluid resuscitation, introduction or increase in vasopressors, decrease or cessation of ultrafiltration. The occurrence of an IHI was recorded 60 minutes (just before starting ultrafiltration if needed), 120 minutes and 240 minutes after starting intermittent hemodialysis.

### Statistical analyses

Continuous variables were described as medians [interquartile ranges] and categorical variables as proportions. Comparisons of proportions between groups were made using Fisher’s exact test. Comparisons of continuous variables between groups were made using Mann–Whitney test. Log-binomial models were used to estimate the relative risk of IHI according to hemodynamics and tissue perfusion variables at T0. Based on the relative risk estimates in the multivariable model, we proposed a simple scoring system by rounding log-coefficients and computed the area under the receiver operating characteristics (AUROC) value. As the same data were used to devise the score and compute its AUROC, we corrected for optimism using the bootstrap [37]; the corrected values were very close to the raw estimates and did not suggest overfitting. We computed the Net Reclassification Index (NRI) to quantify improvement in risk prediction with risk categories defined by above and below the average incidence.

Statistical significance was set at *P* < 0.05. Analyses were made using Prism and R statistical platform, version 3.0.2 (https://cran.r-project.org/).

### Ethical considerations

This observational study was approved by the ethics committee of the French Intensive Care Society (*Société de Réanimation de Langue Française*). All patients and families were informed through a letter that anonymous data would be used for this research and gave their consent.

## Results

### Characteristics of intermittent hemodialysis sessions

Two hundred eleven sessions performed in 88 patients were included in the study, after exclusion of 16 first intermittent hemodialysis sessions performed in extreme emergency. We therefore analyzed 72 first sessions (34% of all sessions) in 72 patients and 139 (66%) later sessions in 88 patients. Characteristics of the included patients are summarized in Table 1. Sessions were performed 8 [3–12] days after ICU admission. One hundred thirty (62%) sessions were performed in patients undergoing mechanical ventilation and 90 (43%) in patients receiving vasopressors. Median length of intermittent hemodialysis sessions was 4 [4-4] hours. Ultrafiltration was performed in 135 (64%) sessions, with a median volume of 2.5 [2.0–3.0] liters (Table 2).

**Table 1:** Characteristics of patients

Number of patients	88
Male	66 (75%)
Age	67 [55–76]
*Comorbidities*	
Hypertension	55 (62%)
Cancer or hematological malignancy	26 (29%)
Diabetes	24 (27%)
Cardiac disease	24 (27%)
Cirrhosis	14 (16%)
Chronic kidney disease	12 (14%)
*Source of ICU admission*	
Ward	42 (48%)
Emergency department	35 (40%)
Home	11 (12%)
*Main diagnosis at ICU admission*	
Acute respiratory failure	24 (27%)
Sepsis	24 (27%)
Acute kidney injury	23 (26%)
Hemorrhagic shock	6 (7%)
Cardiogenic shock	4 (4%)
Coma	2 (2%)
Other	5 (6%)
*SAPS II*	61 [49–74]
*Organ supports during ICU stay*	
Vasopressors	71 (81%)
Non-invasive mechanical ventilation	16 (18%)
Invasive mechanical ventilation	88 (100%)

Quantitative variables are expressed as median (25–75th percentiles) and qualitative variables as number (%)

Chronic kidney disease was defined as glomerular filtration rate < 60 mL/min/1.73 m^2^ according to MDRD formula

**Table 2:** Characteristics of intermittent hemodialysis sessions

	All	First	Later	*P*
Number of sessions	211	72	139	
Number of days since ICU admission	8 [3–12]	3 [2–5]	10 [7–15]	< 0.0001
SOFA score (without renal points)	6 [2–11]	8 [4–13]	4 [1–9]	0.0001
*Organ support therapy at T0*				
Invasive mechanical ventilation	130 (62%)	48 (67%)	82 (59%)	0.30
Vasopressors	90 (43%)	44 (61%)	46 (30%)	0.0001
Norepinephrine	80 (38%)	36 (50%)	44 (32%)	
Epinephrine	10 (5%)	8 (11%)	2 (1%)	
*Cardiovascular SOFA score ≥ 1 at T0*	104 (49%)	48 (67%)	56 (40%)	0.0003
*Tissue perfusion parameters at T0*				
Lactate > 2 mmol/L	49 (23%)	33 (46%)	16 (12%)	< 0.0001
Index capillary refill time ≥ 3 s	38 (18%)	23 (32%)	15 (11%)	0.0003
*Hemodialysis settings*				
Duration (hours)	4 [4–4]	4 [4–4]	4 [4–5]	0.015
Ultrafiltration	135 (64%)	25 (35%)	110 (79%)	< 0.0001
Volume of ultrafiltration (L)	2.5 [2.0–3.0]	2.0 [1.6–3.0]	2.6 [2.0–3.1]	0.10
*Intradialytic hemodynamic instability*	48 (23%)	31 (43%)	17 (12%)	< 0.0001
*Timing of first hemodynamic instability*				0.092
Before first hour	35 (73%)	24 (77%)	4 (24%)	
Between first and second hour	6 (13%)	3 (10%)	1 (6%)	
Between second and fourth hour	7 (14%)	4 (13%)	3 (18%)	
*Therapeutic interventions (not exclusive)*				
Increase in vasopressors	35 (73%)	24 (77%)	11 (65%)	
Fluid administration	15 (31%)	13 (42%)	2 (12%)	
Initiation of vasopressors	7 (15%)	5 (16%)	2 (12%)	
Decrease in ultrafiltration	3 (6%)	0	3 (18%)	
Cessation of ultrafiltration	3 (6%)	0	3 (18%)	

Quantitative variables are expressed as median [25–75th percentiles] and qualitative variables as number (%)

### Intradialytic hemodynamic instability mostly occurred during the first hour of the first intermittent hemodialysis session

IHI occurred in 48 (23%) sessions, mainly within the first hour of intermittent hemodialysis—35 (73%) before ultrafiltration was started if needed. Increase in vasopressors represented the main therapeutic intervention (73%), followed by fluid administration (31%) initiation of vasopressors (15%) and decrease (6%) or cessation of ultrafiltration (6%). IHI was more frequently observed during the first session than in later sessions (43% vs 12%, *P* < 0.0001). Compared to later sessions, first sessions were characterized by higher SOFA score at T0 (8 [4–13] vs 4 [1–9], *P* = 0.0001). Cardiovascular SOFA score ≥1 (67% vs 40%, *P* = 0.0003) and tissue perfusion alterations—index CRT ≥ 3 s (32% vs 11%, *P* = 0.0003) and lactate > 2 mmol/L (46% vs 12%, *P* < 0.0001)—were also more frequently observed at T0 for first sessions (Table 2).

### Cardiovascular SOFA score, index capillary refill time and lactate level at T0 are associated with intradialytic hemodynamic instability

As IHI occurred more frequently during the first intermittent hemodialysis session, we tested whether bedside hemodynamic and tissue perfusion parameters were associated with IHI among the 72 non-emergency first sessions. AUROCs for the cardiovascular SOFA score was 0.67 (95% CI [0.56, 0.78]), it was 0.71 (95% CI [0.59, 0.84]) for index CRT and 0.76 (95% CI [0.66, 0.88]) for lactate. In a log-binomial regression, we found that the following characteristics were more frequent at T0 when intradialytic hemodynamic instability occurred: cardiovascular SOFA score ≥1 (87% vs 51%, *P* = 0.0021, RR = 2.5 95% CI [1.4, 4.5]), index CRT ≥ 3 s (55% vs 15%, *P* = 0.0004, RR = 2.6 95% CI [1.6, 4.3]) and lactate > 2 mmol/L (68% vs 29%, *P* = 0.0018, RR = 3.4 95% CI [1.3, 8.5]) (Table 3).

**Table 3:** Characteristics of first hemodialysis sessions at T0

	IHI	No IHI	*P*
Number of sessions	31	41	
*Organ support therapy at T0*			
Invasive mechanical ventilation	24 (77%)	24 (59%)	0.13
Vasopressors	25 (81%)	19 (46%)	0.0037
*Cardiovascular SOFA score ≥ 1 at T0*	27 (87%)	21 (51%)	0.0021
*Tissue perfusion parameters at T0*			
Lactate > 2 mmol/L	21 (68%)	12 (29%)	0.0018
Index capillary refill time ≥ 3 s	17 (55%)	6 (15%)	0.0004
*Hemodialysis settings*			
Duration (hours)	4 [4-4]	4 [4-4]	0.86
Ultrafiltration	8 (26%)	17 (41%)	0.21

Quantitative variables are expressed as median [25–75th percentiles] and qualitative variables as number (%)

### Bedside SOCRATE (cardiovascular SOFA, index Capillary Refill time and lactATE level) score correlates with the risk of intradialytic hemodynamic instability

**Fig. 1:**
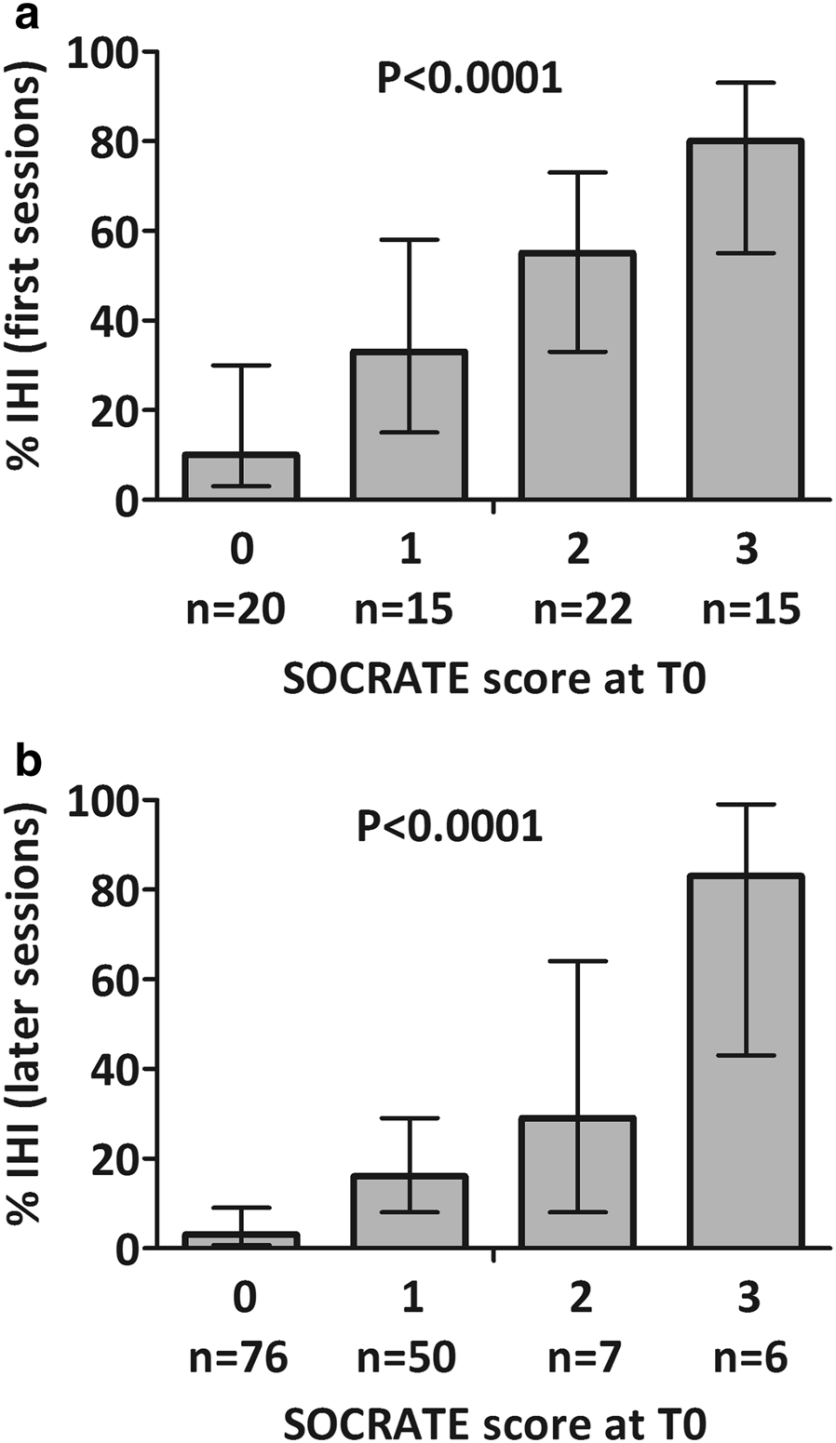
Proportion of intradialytic hemodynamic instability (IHI) during first sessions (**a**) and later sessions (**b**) according to the SOCRATE score (cardiovascular SOFA score ≥ 1, index Capillary Refill time ≥ 3 s and lactATE level > 2 mmol/L). Error bars represent 95% confidence interval

The three relative risks reported above being of the same magnitude, we proposed the SOCRATE score, a simple scoring system derived from the linear predictor of the log-binomial model for IHI occurrence cumulating 1 point for each of cardiovascular SOFA ≥ 1, index Capillary Refill time ≥ 3 s and lactATE level > 2 mmol/L at T0. This score took values between 0 and 3 and split the first sessions in almost 4 equal groups: 28% (*n* = 20), 21% (*n* = 15), 30% (*n* = 22) and 21% (*n* = 15) for SOCRATE scores 0 to 3. IHI incidence was 10% (95% CI [3%, 30%]), 33% (95% CI [15%, 58%]), 55% (95% CI [35%, 73%]) and 80% (95% CI [55%, 93%]) for SOCRATE score of 0, 1, 2 and 3, respectively (Fig. 1), with a relative risk RR = 1.7 (95% CI [1.3, 2.2]) for each additional point in the SOCRATE score. The optimism-corrected AUROC was 0.79 [0.69–0.89] (*P* < 0.0001). NRIs between this model and that including only 2 out of the 3 variables suggested improved risk stratification for the SOCRATE score (Additional file 1: Table S1).

In sessions after the first, a low SOCRATE score was more common (0:55% (*n* = 76) and 1:36% (*n* = 50)) with fewer high scores (2:5% (*n* = 7) and 3:4% (*n* = 6)). The incidence of IHI still increased with the SOCRATE score: 3% (95% CI [0.7%, 9.1%]), 16% (95% CI [8%, 29%]), 29% (95% CI [8%, 64%]), 83% (95% CI [43%, 99%]) for a SOCRATE score of 0, 1, 2 and 3, respectively (*P* < 0.0001) (Fig. 1), with a relative risk of RR = 3.6 [2.4, 4.8] per additional score point. IHI occurrence during a previous session was also associated with an increased risk of IHI (RR = 2.6, 95% CI [1.1, 6.3]). However, adding an extra point to the score in case of previous instability did not improve the NRI with respect to the SOCRATE score alone.

## Discussion

In a prospective study including 211 hemodialysis sessions performed in a mixed ICU population, we found that IHI mostly occurred at the initiation of intermittent hemodialysis—during the first hour of the first session—despite the absence of fluid removal. A cardiovascular SOFA score ≥1, and two tissue hypoperfusion markers, index CRT ≥ 3 s and lactate level > 2 mmol/L, were associated with the occurrence of IHI. In addition, the risk of IHI increased with the number of abnormal parameters. A bedside score combining these three parameters, named SOCRATE score (cardiovascular SOFA score ≥ 1, index CRT ≥ 3 s and lactATE > 2 mmol/L), improved risk stratification with a good accuracy.

Intermittent hemodialysis is a key support therapy in ICU. Despite protocol-based optimization [5], IHI remains a frequent issue in critically ill patients. IHI incidence varies from one study to another because of discrepancies in IHI definition and in preventive protocols [5–11, 38, 39]. In our cohort, using a pragmatic definition of IHI, blood pressure drop requiring a medical intervention, we found an incidence of 23%. Using the same definition, Monnet et al. [9] found higher incidence (33%), but all patients underwent fluid removal.

IHI mainly occurred during the first hour of treatment in the absence of ultrafiltration. During intermittent hemodialysis, due to partial redistribution of blood volume from the intravascular compartment to the extracorporeal circuit, mild hypovolemia is constantly induced even in the absence of ultrafiltration. However, blood pressure does not systematically decrease because of counter-regulatory mechanisms such as tachycardia, increased cardiac contractility, plasma refilling and peripheral vasoconstriction [16, 40]. One could speculate that pre-existing alterations of macro- and microcirculation could interfere with these cardiovascular adaptation mechanisms and could promote IHI. Pre-existing microvascular endothelial dysfunction, commonly observed in ICU patients [19–23], might alter such adaptive vasoconstriction [20], promoting IHI. Hemodialysis, by itself, could also alter endothelial function. Meyer et al. [17] showed an increase in “vasculotoxic” cell-free hemoglobin in the plasma of chronically dialyzed patients during intermittent hemodialysis. In addition, microbubbles generated in the circuit could damage endothelium glycocalyx, triggering activation of coagulation, platelets, neutrophils and promoting oxygen reactive species release [41–43].

The evaluation of endothelial dysfunction at the bedside is very challenging [24, 25], but its consequences in term of tissue hypoperfusion, could be assessed more easily. We speculated that tissue hypoperfusion parameters reflect microvascular endothelial dysfunction and we investigated two easy-to-use parameters, rapidly available at the bedside, the lactate level and the index CRT. Lactate level, widely used in ICU, is inversely correlated with sublingual microvascular perfusion [44, 45] and is predictive of ICU mortality in septic shock patients [28]. CRT measurement correlates with the pulsatility index, a surrogate ultrasound-derived parameter that reflects vascular tone of visceral organs [46] and with objective parameters of tissue perfusion, such as tissue oxygen saturation in patients with septic shock [47]. CRT is associated with hyperlactatemia and severity of critical illness addressed by SOFA score [48] and predicts 14-day mortality in patients with septic shock independently of SOFA score [34].

Here, we found for the first time that increased index CRT and hyperlactatemia were associated with IHI occurrence, as well as impaired global hemodynamics defined by a cardiovascular SOFA score ≥1. More interestingly, we found a cumulative predictive effect of these parameters similar to that we observed in the setting of severe pulmonary embolism [49]. Combining them in a score which can be quickly and easily calculated at the bedside could be helpful to improve risk stratification. IHI was rare in the absence of macro- and micro-circulatory disorders (10% in first sessions, 3% in all the sessions) and increased progressively with the number of abnormal parameters reaching 80% when all three markers were present. In the absence of emergency criteria, intermittent hemodialysis initiation might be reconsidered in patients with higher SOCRATE score, indicating a high risk of IHI. Such a hypothesis is supported by the fact that optimal timing to initiate RRT in critically ill patients is still uncertain [4, 13, 14]. Recent trials demonstrated that delaying RRT in the absence of life-threatening complications of AKI does not impair survival and allows some patients to avoid RRT [4, 13]. These results plead in favor of a reasoned strategy balancing risks and benefits for initiating RRT in ICU patients.

Moreover, if intermittent hemodialysis indication is retained, SOCRATE score might be helpful for clinicians to identify patients who may benefit from a therapeutic intervention aiming at optimizing hemodynamic status before intermittent hemodialysis initiation in order to decrease the occurrence of IHI.

Our study has several limitations. It is a monocentric study, and the results need to be confirmed in a multicenter study including a larger population. Nevertheless, we analyzed a large number of intermittent hemodialysis sessions in a mixed non-selected medical ICU population. We did not include patients with dark skin because CRT was difficult measure. It would be interesting to test the prognosis value of a score combining lactate and cardiovascular SOFA alone, or in association with other validated clinical parameters of peripheral hypoperfusion such as central-to-toe temperature difference [50]. As we could not include sessions performed in extreme emergency because data could not be recorded before the beginning of intermittent hemodialysis, IHI incidence was probably underestimated. Finally, our study focused on patients receiving intermittent hemodialysis. Future studies also including patients receiving continuous RRT are needed to test whether SOCRATE score could help to identify patients at risk of hemodynamic instability whatever the modality of RRT.

## Conclusions

The risk of intradialytic hemodynamic instability is maximal at intermittent hemodialysis initiation, during the first hour of the first sessions, even in the absence of ultrafiltration. Cardiovascular SOFA ≥ 1, hyperlactatemia and increased index capillary refill time are associated with an increasing risk of IHI and combining these parameters (SOCRATE score) improves risk stratification. A multicenter study would be useful to confirm these results, paving the way for a future trial evaluating whether a therapeutic strategy based on SOCRATE score before hemodialysis initiation may limit IHI.

## Supplementary information

**Additional file 1: Table S1.** Comparison of SOCRATE score with 2-variable models *AIC* Akaike information criterion, *AUROC* area under the receiver operating characteristics, *CRT* capillary refill time, *NRI* net reclassification index, *SOFA* sequential organ failure assessment.

## Data Availability

The datasets used and/or analyzed during the current study are available from the corresponding author on reasonable request.

## Abbreviations

AKI: Acute kidney injury
CRT: Capillary refill time
ICU: Intensive care unit
IHI: Intradialytic hemodynamic instability
RRT: Renal replacement therapy
SOFA: Sequential Organ Failure Assessment
