# Supplementary material for: Bedside prediction of intradialytic hemodynamic instability in critically ill patients: the SOCRATE study
Source: Ann Intensive Care. 2020 Apr 22;10:47. doi: 10.1186/s13613-020-00663-x (PMC7176798; doi:10.1186/s13613-020-00663-x)
Supplement: Supplementary file 1 — Additional file 1: Table S1. Comparison of SOCRATE score with 2-variable models AIC Akaike information criterion, AUROC area under the receiver operating characteristics, CRT capillary refill time, NRI net reclassification index, SOFA sequential organ failure assessment. [file 13613_2020_663_MOESM1_ESM.docx]

**Table S1. Comparison of SOCRATE score with 2-variable models**

|  | 3-variable SOCRATE score  (lactate >2 mmol/L,  index CRT ≥3 seconds, cardiovascular SOFA score ≥1) | 2-variable score  (lactate >2 mmol/L,  index CRT ≥3 seconds) | 2-variable score  (index CRT ≥3 seconds, cardiovascular SOFA score ≥1) | 2-variable score  (lactate >2 mmol/L, cardiovascular SOFA score ≥1) |
| --- | --- | --- | --- | --- |
| **First sessions (n=72)** | | | | |
| AIC | 82.7 | 84.4 | 84.4 | 88.5 |
| AUROC | 0.79 [0.69, 0.89] | 0.77 [0.66, 0.87] | 0.77 [0.66, 0.87] | 0.74 [0.63, 0.85] |
| ΔAUROC P-value (deLong’s test) | - | 0.36 | 0.4 | 0.06 |
| NRI (below/above average 43% | - | 0.02 [-0.07, 0.10] | 0.06 [-0.12, 0.26] | 0.11 [-0.02, 0.27] |
| **All sessions (n=211)** | | | | |
| AIC | 166.7 | 173.1 | 176.2 | 177.6 |
| AUROC | 0.84 | 0.80 | 0.81 | 0.80 |
| ΔAUROC P-value (deLong’s test) | - | 0.05 | 0.05 | 0.03 |
| NRI (below/above average 23%) |  | -0.001 [-0.09, 0.07] | 0.094 [0.0, 0.20] | 0.07 [-0.06, 0.20] |

Abbreviations: AIC: Akaike Information Criterion; AUROC: Area Under the Receiver Operating Characteristics; CRT: capillary refill time; NRI: Net Reclassification Index; SOFA: Sequential Organ Failure Assessment
